# Missed Opportunities for Childhood Immunization in Pakistan: Failure to Vaccinate Despite Health System Contact

**DOI:** 10.12688/gatesopenres.16388.1

**Published:** 2026-06-23

**Authors:** Areesh Fatmee, Zia un Nisa, Muhammad Ibrahim, Aisha Irum, Ayesha Khan, Adnan Ahmad Khan

**Affiliations:** 1 Research and development solutions, Islamabad, Pakistan, 44000, Pakistan; 2 Akhter Hameed Khan Foundation, Islamabad, 44000, Pakistan; 3 Ministry of National Health Services, Regulations and Coordination (MoNHSRC), Islamabad, 44000, Pakistan

**Keywords:** Missed dose, zero dose, immunization, missed opportunities for vaccination (MOV), public vs. private healthcare

## Abstract

Despite global gains in vaccination coverage, Pakistan continues to lag in achieving universal immunization, with vaccine-preventable diseases (VPDs) contributing to approximately 15% of all deaths annually. We explored missed opportunities for vaccination (MOV) when eligible children failed to receive vaccines when their mothers visited healthcare facilities for unrelated medical needs. Secondary data analysis was conducted using the Pakistan Social and Living Standards Measurement Survey (PSLM) 2019-20, comprising 195,000 households and 103,464 eligible children. MOV was defined as the failure to administer an age-appropriate dose of either pentavalent (Penta-1, 2, 3) or measles (Measles-1, 2) vaccines during a maternal health visit. Weighted multivariate binomial logistic regression was used to explore the association between maternal visits to private healthcare facilities and MOV, adjusting for sociodemographic variables. Geospatial analysis was used to visualize district-level disparities using ArcGIS. Approximately 25% of children aged 1-5 years missed at least one dose of pentavalent or measles vaccination with regional variations. The rates of missed doses were similar for the first, second, and third antigen doses. Rural areas, male children, and households with uneducated mothers had more missed vaccinations, while missed opportunities were more common when mothers visited private healthcare facilities, particularly in urban settings (adjusted Odds Ratio (OR): 1.563 for Penta-1, Confidence Interval (CI): 1.519-1.608) where the private sector predominates and because the public sector accounts for nearly all vaccinations. However, since nearly all vaccinations are in the public sector and the bulk of healthcare seeking is in the private sector, opportunities to vaccinate missed children are lost. Policy and technological solutions that can identify and refer to these children for vaccination can close the national gap in universal vaccination.

## Introduction

Since the launch of the Expanded Program for Immunization in 1974, national immunization programs have used the platform to significantly improve global vaccination coverage and reduce the incidence of vaccine-preventable diseases (VPD). ^1^ Despite these efforts, global coverage, as measured by the third dose of pentavalent vaccine (Penta-3), has plateaued at 86% since 2010. ^2^^,^^3^ Resource constraints in low-to middle-income countries (LMICs) often lead to unreliable health delivery systems owing to the limited supply of trained health personnel, vaccines, and infrastructure. ^4^ This results in a limited coverage of vital vaccines. A key concept in addressing gaps in coverage is to identify missed opportunities for vaccinations (MOV), where children do not receive necessary vaccines during healthcare visits for themselves or by their mothers. ^5^ Addressing these factors can help improve the coverage gaps in LMIC. ^6^

In contrast to scaling-up campaigns such as immunization, when there has been low coverage, reaching the last mile or the last few percent of individuals is often more difficult and often hinges on recognizing and addressing subtle challenges in specific local contexts and populations. ^7^^,^^8^ However, sociocultural, logistical, and systemic gaps may also confound campaigns and vary from region to region. These challenges have drawn suggestions for enhanced surveillance, ^8^ localization of decision-making, and higher community engagement, ^9^ and improving collaboration between the public and private healthcare sectors. ^10^^,^^11^ These are addressed in the Global Vaccine Action Plan (GVAP), which seeks to reach ≥90% national coverage for all vaccines by 2020 for all countries by engaging communities and civil society organizations (CSO) and the private sector. ^12^^,^^13^

Pakistan has the third highest number of under-vaccinated children globally. ^14^^,^^15^ VPD accounts for nearly two-thirds of all infant deaths, which in turn account for nearly 22% of all deaths in Pakistan. ^16^ Critical gaps in vaccination coverage have led to frequent outbreaks, including one for diphtheria in Khyber Pakhtunkhwa (KP), which led to over 1,900 cases and 200 deaths in 28 of the 37 districts in the province. ^17^ There have been other large-scale measles outbreaks in 2012-13 and 2016-18 across the country, and more recent ones in Sindh in 2022 and KP in 2023. ^18^^–^^20^ Although vaccination rates have improved, with complete vaccination coverage increasing from 54% in 2012-13 to 76% nationwide in 2020 among children aged 12-23 months, missed vaccinations persist and continue to pose a substantial threat. ^21^ Therefore, the lack of an in-depth evaluation of the MOV burden during routine service delivery in Pakistan remains unexplored. Our analysis is conceptually informed by the WHO framework on missed opportunities for vaccination, which considers service-level, provider, and caregiver barriers during health encounters. We adapted this by focusing on maternal healthcare visits as critical interaction points where child vaccination status may be overlooked. ^22^ This study assessed the prevalence of MOV and bio- and socio-demographic factors that affect MOV for Pentavalent (Penta-1,2,3) and Measles (Measles-1,2) vaccinations for children aged 1-5, whose mothers attended private or public healthcare facilities for medical care.

## Methodology

### Study design and Data source

A secondary data analysis was conducted using the Pakistan Social and Living Standards Measurement Survey (PSLM) 2019-20. Data on sociodemographic factors were extracted to contextualize the reasons for MOVs. The PSLM survey provides biannual national-level statistics that help governmental agencies, stakeholders, and non-governmental organizations monitor health progress using key indicators. Based on the variation in population characteristics in the distribution of urban and rural populations, the availability of resources, cost, and disability coverage. A sample of 195,000 households from 6500 sample areas was taken, and 103,464 children were eligible for immunization. Final regression analysis was conducted on approximately 2500 above mothers who visited healthcare facility. Ethical approval was not required for the study.

### Sample and Participants

The analysis included children under 12 months who were excluded due to the standard administration of the measles vaccine at 12 months and the absence of precise birthdates, ensuring the inclusion of only eligible participants. Children with zero-dose status were excluded from the primary analysis to focus specifically on MOV occurring during identifiable healthcare encounters, consistent with the WHO MOV framework. Although Pakistan’s immunization system includes outreach modalities such as door-to-door campaigns, mobile teams, and community health workers, the PSLM dataset dose not capture the timing, or nature of these contacts. Consequently, it is not possible to ascertain whether zero-dose children experience a missed opportunity during a documented health interaction. Their exclusion therefore reflects a methodological constraints of the dataset rather than an assumption of no health system contact, which is acknowledged as a study limitation.

### Outcome variable

MOV was defined as failure to receive an age-appropriate dose of either the pentavalent or measles vaccine despite the mother’s visit to a healthcare facility. Defining MOV as a binary variable involved assessing whether children aged 1-5 missed at least one dose of either pentavalent or measles vaccine despite maternal contact with any healthcare facility. The variable was coded as 1 if the dose was unadministered despite contact and 0 otherwise, based on recall or documented vaccination records. Separate variables were created for each vaccine dose (penta-1, penta-2, penta-3, measles-1, and measles-2). Table 1 shows Pakistan’s expanded program on immunization (EPI) schedule for pentavalent and measles vaccines.

**Table 1.** EPI schedule for Pentavalent and Measles vaccination in Pakistan.

Vaccine	Doses	Age of child
Pentavalent vaccine (Diphtheria/Pertussis/Tetanus (DPT)+ Hemophilus influenzae (Hib) +Hepatitis B)	3	Penta-1 at 6 weeks
		Penta-2 at 10 weeks
		Penta-3 at 14 weeks
Measles	2	Measles-1 at 9 months
		Measles-2 at 12 months

### Predictor variable

We used reports of contact with healthcare facilities through maternal visits as a predictor variable. The private sector included clinics, dispensaries, or hospitals, and public sector facilities such as public dispensaries, hospitals, Basic Health Units (BHU), Rural Health Centers (RHC), visits to Lady Health Visitors (LHV), and Lady Health Workers (LHW). Visits were coded as a binary variable: 1 for the private sector and 0 otherwise. Our evaluation used maternal healthcare access data due to popular evidence of infrequent vaccinations during sick child visits, the influence of false contraindications, and providers’ widespread failure to screen for vaccinations. ^23^ Based on prior literature and their availability in the PSLM 19-20 database, the regression model incorporated several determinant variables, including socio-demographic variables (region, child age, child sex, total household members, household head sex, maternal age, maternal formal education, maternal employment status, and wealth index).

### Analysis

The prevalence of MOVs for each dose of pentavalent and measles vaccines was calculated in children aged 1–5 years. Among children identified with MOV, the proportion of mothers who consulted healthcare facilities due to sickness or injury within the last two weeks was marked. These proportions were further stratified by the types of healthcare facilities consulted and determinant variables. To visually represent these findings, maps showing the proportions of missed vaccination opportunities for each dose of pentavalent and measles vaccines across Pakistan were generated using ArcGIS software (V 10.8.2).

Those who had received at least one vaccination dose but encountered MOVs during subsequent opportunities were the focus of the study, whereas children with zero dose status were excluded. Potential confounders were selected a priori based on existing literature on childhood immunization and the WHO conceptual framework for MOV. Variables were included if they were plausibly associated with both healthcare-seeking behavior and vaccination outcomes, and were available in the PSLM dataset. These included child characteristic (age, sex), household characteristics (household size, sex of household head and wealth index), maternal characteristics (age, education, employment status), and context factors (urban-rural residence and province). All regression models adjusted for the same set of covariates to ensure comparability across vaccine doses. No data-driven stepwise procedures were used. For multivariate analysis, weighted, multivariable binomial logistic regression was used to measure the association between maternal visits to private healthcare facilities and missed opportunities for pentavalent (Penta-1, Penta-2, and Penta-3) and measles (Measles-1 and Measles-2) vaccinations. The model was adjusted for potential confounders to yield adjusted odds ratio estimates. A sub-analysis was conducted for each province to assess disparities among the provinces. Measures of association are presented as adjusted odds ratios (aORs) with 95% confidence intervals (CI). Mother’s reported healthcare facility issues (i.e. staff cooperation, waiting time, cleanliness, medicine availability, doctors availability, treatment cost, treatment failure) were included as binary covariates in the national-level regression model and adjusted for alongside sociodemographic factors. All analyses were performed using STATA version 17 (College Station, Texas, USA).

## Results

### Missed vaccinations

**Figure 1.**
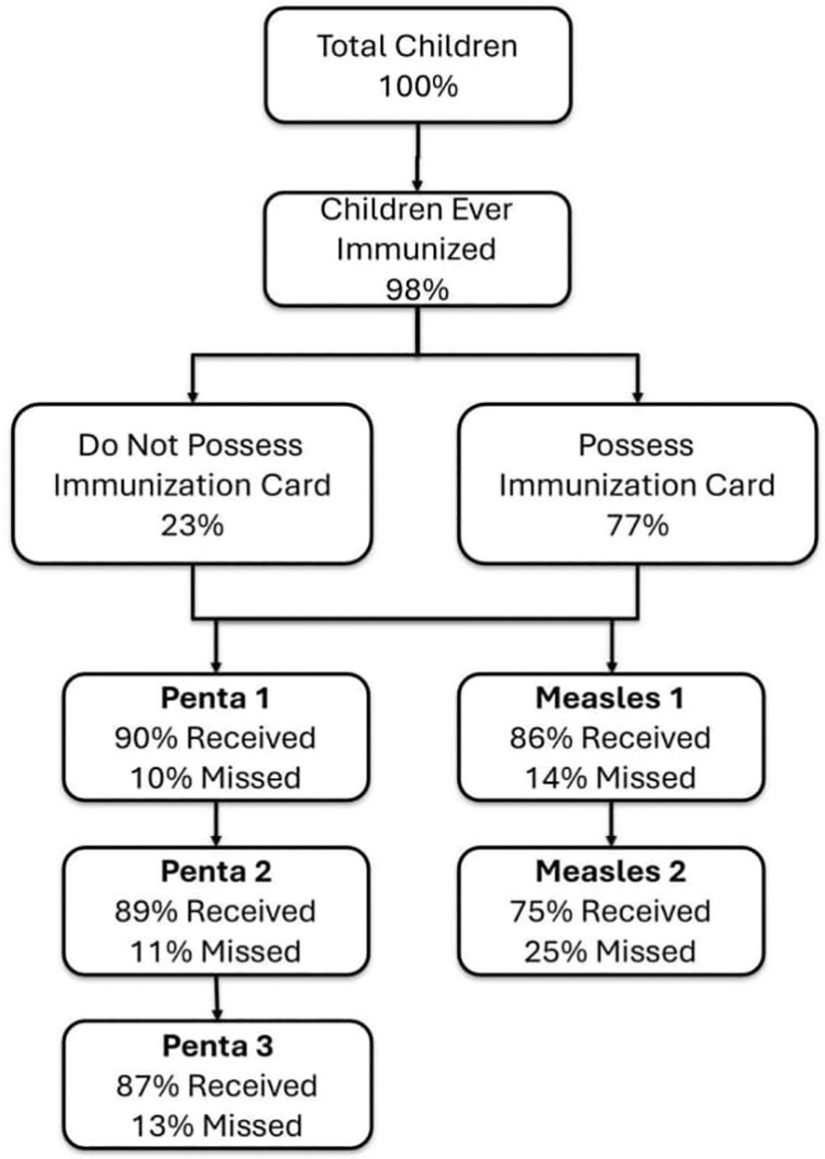
Flow-Chart Analyzing Distribution of Children Aged 1-5 for Assessment of Missed Opportunities of Vaccination, 2019-2020.

We found that 26% of children aged 1-5 missed at least one dose of measles, and 13% had missed a pentavalent vaccine ( Figure 1).

At the national level, missed vaccine rates range from 9.7% to 25%, with the highest rates in Balochistan, ranging from 29.5% to 43.1% for specific vaccines, and the lowest in Punjab, where the range is between 3.2% and 18.6%. Missed vaccinations were higher in rural than in urban areas (11.2 to 24.9%), and for children of uneducated mothers (17.1% to 30.9%), and among those visiting private facilities. Although point estimates suggested higher missed opportunities among male children (10.2 to 25.4% for boys vs. 9.3 to 24.5% for girls), and male-led households (10–25.4% vs. 5.8–19.2%), the confidence intervals overlapped, indicating that these differences should be interpreted cautiously.

**Figure 2.**
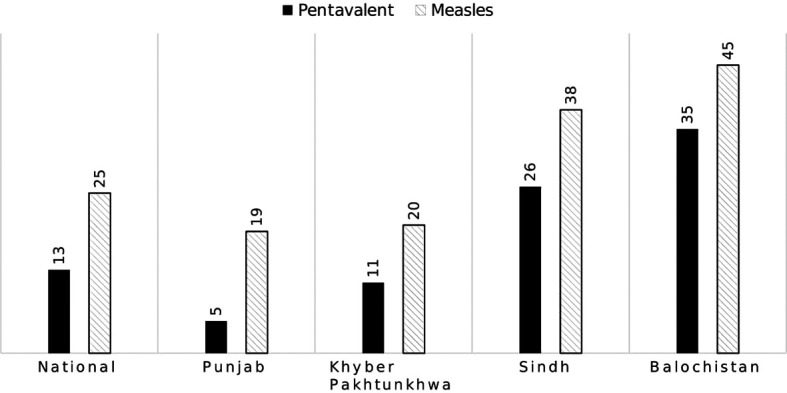
All Missed Vaccinations for Pentavalent and Measles Doses on National & Provinces of Pakistan in 2019-2020.

At the national level, pentavalent vaccines were missed by 13% and measles by 25% ( Figure 2), with considerable interprovincial variation. There are always more children who miss measles than the pentavalent vaccine.

**Figure 3.**
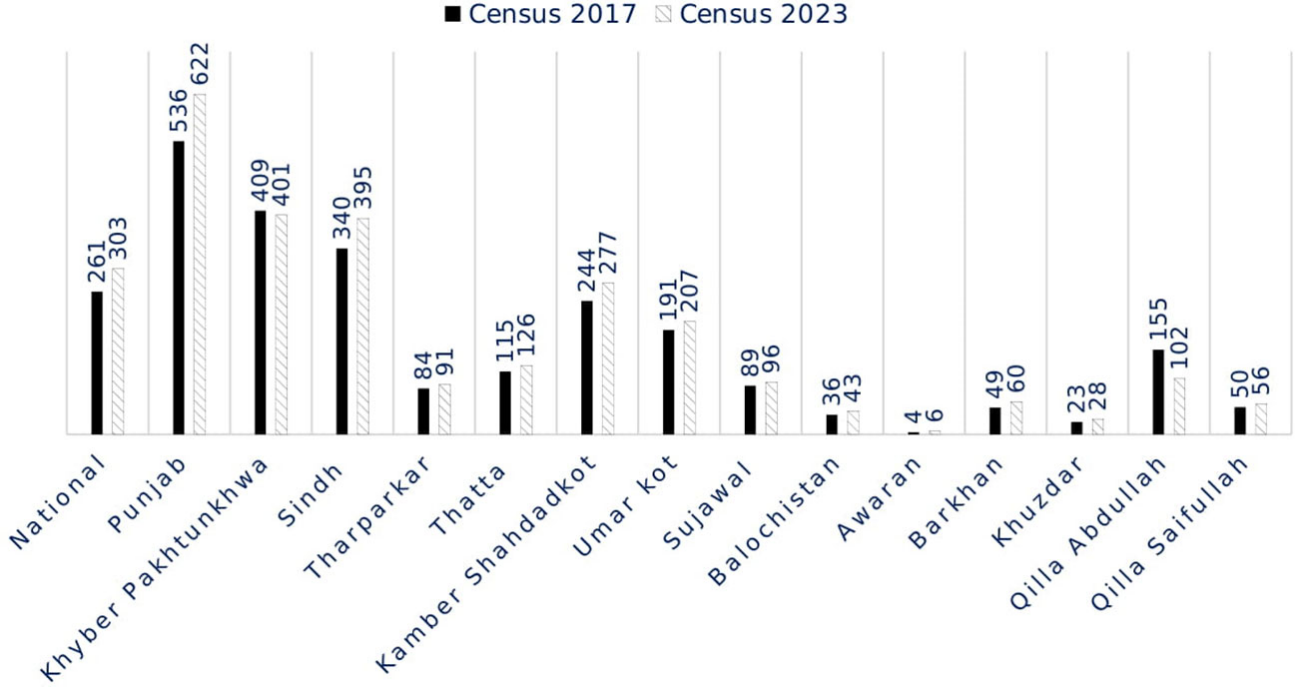
Population Density per Square KM of Pakistan at the National, Provincial and District Level from Census 2017 & 2023.

The number of missed vaccinations was higher in sparsely populated areas. Balochistan has the least dense population and the highest missed rates at 35.4% for pentavalent and 45.4% for measles, followed by Sindh with 26.1% for pentavalent and 38.5% for measles. In contrast, Punjab and KP, with denser populations, had the lowest missed rates. At the district level, in Balochistan, very sparsely populated Khuzdar had 83.8% pentavalent and 88.4% measles missed cases, while Awaran had 80.9% and 89% missed cases, respectively. In Sindh, Tharparkar reported 80.2% for pentavalent and 87.4% for measles, while Kamber Shahdabkot reported 63.2% and 71.5%, respectively ( Figure 3).

**Figure 4.**
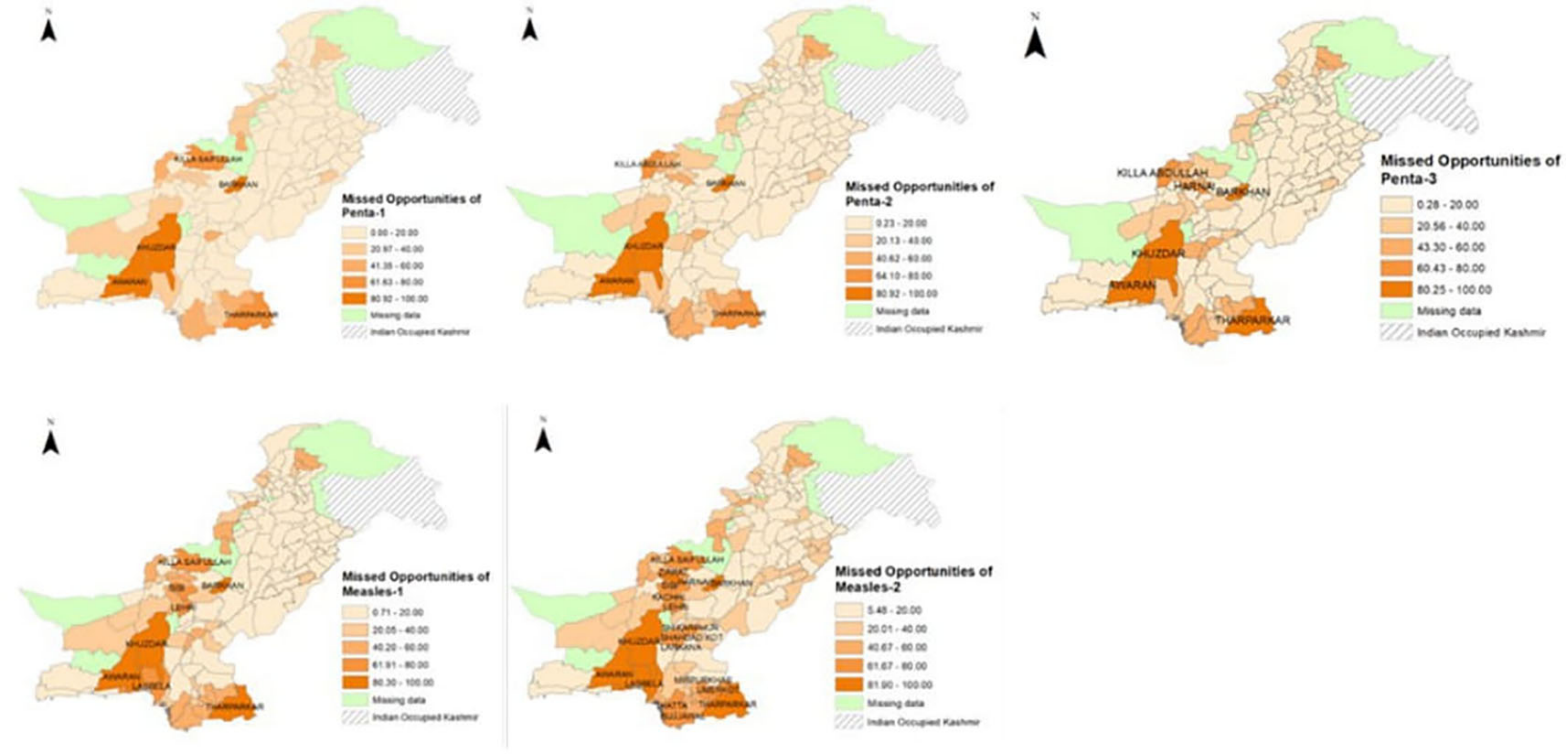
Geospatial Analysis of Missed Opportunities for Vaccination (MOVs) for Pentavalent and Measles Doses in Districts of Pakistan in 2019-2020.

This population-missed vaccination relationship was visualized using heat maps ( Figure 4). The districts of Awaran (Penta-1 = 80.9%, Penta-2 = 80.9%, Penta-3 = 80.9%, Measles-1 = 80.5%, Measles-2 = 88.4%), Barkhan (Penta-1 = 81.2%, Penta-2 = 81.6%, Penta-3 = 81.3%, Measles-1 = 82.4%, Measles-2 = 83.8%), Khuzdar (Penta-1 = 83.8%, Penta-2 = 83.8%, Penta-3 = 83.9%, Measles-1 = 83.8%, Measles-2 = 88.3%), Qilla Saifullah (Penta-1 = .61.6%, Penta-2 = 64.1%, Penta-3 = 67.01%, Measles-1 = 61.9%, Measles-2 = 68.5%), and Qilla Abdullah (Penta-1 = 54.8%, Penta-2 = 54.9%, Penta-3 = 55.63%, Measles-1 = 49.9%, Measles-2 = 52.7%) in Balochistan along with Tharparkar (Penta-1 = 71.1%, Penta-2 = 76.2%, Penta-3 = 82.3%, Measles-1 = 80.3%, Measles-2 = 87.4%) in Sindh, respectively. The spatial distributions of pentavalent and measles were identical in various districts of Pakistan.

### Missed opportunities to vaccinate

When a mother presents to a healthcare provider, there is an opportunity to ask for and catch up with missed doses of vaccines for her children. However, if the mother visits facilities that do not offer vaccinations, such opportunities are consistently lost. We found that 10% of mothers had children with a missed dose of the pentavalent vaccine (Penta 1). Of these, 91% had visited a healthcare provider. Nationally, the odds of missing the first dose of the pentavalent vaccine (Penta-1) were higher if a private facility was visited (OR: 1.364, CI: 1.337, 1.391) compared to those visiting public facilities ( Table 3). Missed opportunities vary across the provinces. KP had the most missed opportunities for the third dose of pentavalent vaccine (Penta-3) (OR: 2.232, CI: 2.119, 2.351). In contrast, Sindh (OR: 0.555, CI: 0.539, 0.571) had fewer odds of missing penta-3 if the mother had visited a private facility compared to a public facility. For measles vaccination, Balochistan had the highest burden of Measles-2 in the context of private healthcare setups (OR: 1.360, CI: 1.271, 1.455). For Measles-2, Punjab demonstrated the lowest odds of missed vaccinations compared to the other provinces (OR: 0.697, CI: 0.682, 0.712). Individual provincial-level regression analyses are presented in Annexure Tables 1– 4.

**Table 2.** Descriptive Statistics of Missed Vaccinations Among Children under 5 of Age Pakistan Social and Living Measurements Survey 2019-20.

Determinants	Missed Penta-1 %	Missed Penta-2 %	Missed Penta-3 %	Missed Measles-1 %	Missed Measles-2 %
**National**	9.7	11.2	12.8	13.9	24.9
**Province**					
KP	8.3	9.1	10.2	11.8	19.8
Punjab	3.2	4.3	4.9	6.6	18.6
Sindh	20.8	23.9	26.8	27.1	39.3
Balochistan	29.5	30.7	33.5	34.9	43.1
**Region**					
Rural	11.2	12.4	13.4	14.8	24.9
Urban	6.6	8.8	10.7	12.	25.0
**Child sex**					
Male	10.2	11.7	13.2	14.5	25.4
Female	9.3	10.7	11.9	13.3	24.5
**Child age**					
1	8.6	11.3	12.6	14.0	52.9
2	9.9	11.0	12.8	14.3	19.8
3	9.6	10.8	11.9	13.6	16.8
4	10.5	11.8	12.9	13.9	17.2
**Household head sex**					
Male	10.0	11.6	12.9	14.3	25.4
Female	5.8	6.8	7.7	9.2	19.2
**Wealth Quintile**					
Poorest	19.8	21.2	22.5	23.9	33.2
Poor	8.9	10.1	11.4	12.8	23.2
Lower Middle	6.6	7.7	8.7	10.4	21.1
Upper Middle	4.5	6.0	7.1	8.6	20.3
Richest	4.7	6.8	8.8	9.4	23.3
**Maternal Characteristics (at least one dose was missed)**					
Mothers who reported at least one missed dose for their children	10.0	11.5	12.9	14.2	24.8
Mothers sick/injured in last 2 weeks	5.3	5.6	5.7	5.3	5.1
Mothers visiting any healthcare facility in last 2 weeks	90.6	91.3	89.8	89.4	91.4
Visiting Private facilities	50.0	47.6	46.7	47.5	51.2
Visiting Public facilities	42.8	44.8	45.5	44.7	42.5
Visiting non-professional facilities	7.2	7.6	7.8	7.8	6.79
**Mother Formal Education**					
Uneducated	17.1	18.6	19.9	21.6	30.9
Primary	5.7	6.9	8.2	9.6	21.0
Secondary	4.5	6.1	7.5	8.6	20.8
Higher	2.9	4.0	5.3	5.9	18.6
**Mother Employment**					
Employed	11.6	12.7	14.0	14.8	24.5
Unemployed	13.3	16.5	19.9	23.7	13.9
Not in labor force	9.8	11.2	12.6	13.9	24.7

**Table 3.** Logistic Regression of Missed Opportunities of Pentavalent and Measles Vaccination Among Children Aged 1–4 Years at National and Provincial Level in Pakistan (2019-20).

Type of facility [base: visited public healthcare facilities]	Penta-1	Penta-2	Penta-3	Measles-1	Measles-2
**Visited private healthcare facilities**					
National	1.364* [1.337,1.391]	1.118* [1.098,1.138]	1.060* [1.042,1.078]	1.027* [1.010,1.044]	0.922* [0.909,0.935]
KP	2.739* [2.586,2.901]	2.115* [2.006,2.231]	2.232* [2.119,2.351]	1.301* [1.243,1.362]	1.118* [1.076,1.163]
Punjab	1.788* [1.706,1.875]	1.062* [1.023,1.102]	0.848* [0.819,0.878]	1.056* [1.022,1.091]	0.697* [0.682,0.712]
Sindh	0.814* [0.788,0.841]	0.701* [0.680,0.723]	0.555* [0.539,0.571]	0.649* [0.629,0.669]	0.716* [0.696,0.737]
Balochistan	1.211* [1.126,1.303]	1.143* [1.066,1.227]	1.417* [1.324,1.517]	1.044 [0.974,1.120]	1.360* [1.271,1.455]

Note: *p < 0.05 *Controlled for confounders in logistic regression. All models adjusted for child age, and sex, maternal age, maternal education, maternal employment status, household size, household head sex, wealth index, region, and province.

**Table 4.** Logistic Regression of Missed Opportunities of Pentavalent and Measles Vaccination Among Children Aged 1-4 Years at National Level in Pakistan (2019-20).

Type of facility [base: visited public healthcare facilities]	Penta-1	Penta-2	Penta-3	Measles-1	Measles-2
National (Visited private healthcare facilities)	1.628* [1.593,1.664]	1.302* [1.277,1.328]	1.188* [1.166,1.210]	1.151* [1.131,1.172]	0.982* [0.968,0.997]
**Region [Base: Rural]**					
Urban	1.636* [1.589,1.685]	1.808* [1.761,1.856]	2.063* [2.013,2.114]	2.105* [2.056,2.156]	1.590* [1.561,1.619]
**Household Head Gender [Base: Male]**					
0	0.523* [0.488,0.562]	0.661* [0.625,0.700]	0.659* [0.624,0.695]	0.609* [0.577,0.643]	0.549* [0.527,0.572]
Total children in household	1.065* [1.056,1.075]	1.050* [1.041,1.059]	0.967* [0.960,0.974]	1.021* [1.014,1.028]	0.955* [0.950,0.960]
**Age of Child [Base:1 year]**					
2	1.089* [1.056,1.123]	0.703* [0.684,0.722]	0.900* [0.877,0.922]	0.765* [0.746,0.784]	0.213* [0.209,0.217]
3	1.153* [1.325,1.404]	0.766* [0.747,0.786]	0.768* [0.749,0.787]	0.763* [0.745,0.781]	0.165* [0.162,0.168]
4	1.364* [1.325,1.404]	0.905* [0.882,0.928]	1.043* [1.019,1.069]	0.962* [0.939,0.985]	0.190* [0.187,0.194]
**Child Gender [Base: Female]**					
Male	1.011 [1.992,1.031]	0.981* [0.964,0.999]	0.948* [0.932,0.964]	0.997 [0.981,1.014]	1.149* [1.133,1.165]
Total members of household	0.875* [0.870,0.881]	0.861* [0.857,0.866]	0.897* [0.893,0.901]	0.900* [0.896,0.901]	0.948* [0.946,0.951]
Maternal Age	1.007* [1.005,1.009]	0.997* [0.995,0.999]	0.989* [0.988,0.991]	0.987* [0.986,0.989]	0.983* [0.982,0.984]
**Maternal employment status [base: employed]**					
Unemployed	2.506* [2.383,2.636]	2.801* [2.674,2.935]	3.834* [3.665,4.011]	3.355* [3.208,3.509]	2.100* [2.015,2.190]
Not in labor force	0.832* [0.812,0.853]	0.809* [0.790,0.828]	0.861* [0.841,0.881]	0.850* [0.831,0.870]	0.847* [0.830,0.863]
**Maternal formal education [Base: uneducated]**					
Primary education	0.763* [0.740,0.786]	0.631* [0.613,0.649]	0.719* [0.701,0.738]	0.817* [0.796,0.838]	1.058* [1.036,1.079]
Secondary education	0.494* [0.478,0.510]	0.534* [0.519,0.549]	0.518* [0.504,0.532]	0.666* [0.648,0.683]	0.783* [0.767,0.800]
Higher education	0.405* [0.382,0.429]	0.435* [0.414,0.458]	0.323* [0.307,0.339]	0.382* [0.363,0.402]	0.432* [0.417,0.448]
**Wealth index [Base: Poorest]**					
Lower middle	0.295* [0.286,0.305]	0.423* [0.411,0.435]	0.369* [0.359,0.380]	0.514* [0.501,0.528]	0.580* [0.576,0.593]
Middle	0.347* [0.335,0.359]	0.375* [0.363,0.387]	0.320* [0.310,0.330]	0.381* [0.369,0.393]	0.696* [0.679,0.713]
Upper middle	0.227* [0.218,0.236]	0.265* [0.255,0.275]	0.273* [0.263,0.282]	0.290* [0.281,0.300]	0.531* [0.517,0.545]
Richest	0.358* [0.342,0.375]	0.413* [0.396,0.430]	0.422* [0.406,0.439]	0.381* [0.366,0.396]	0.603* [0.584,0.622]
**Issues about Healthcare Facilities that lead to MOV**					
Not cooperative staff	2.138* [2.035,2.247]	2.130* [2.036,2.229]	1.795* [1.716,1.878]	1.837* [1.758,1.919]	1.794* [1.724,1.867]
Lack of cleanliness	2.435* [2.249,2.637]	3.201* [2.960,3.463]	8.740* [8.066,9.469]	8.584* [7.939,9.282]	7.496* [6.957,8.076]
Long waiting time	1.727* [1.648,1.809]	1.753* [1.680,1.829]	2.034* [1.954,2.117]	1.498* [1.438,1.561]	1.025 [0.985,1.066]
Expensive treatment	0.997 [0.965,1.031]	1.195* [1.160,1.231]	1.237* [1.202,1.272]	1.373* [1.336,1.411]	0.967* [0.944,0.991]
Doctor not available	1.191* [1.101,1.288]	2.178* [2.049,2.314]	2.233* [2.109,2.364]	2.592* [2.453,2.739]	1.353* [1.286,1.425]
Medicine not available	2.641* [2.536,2.751]	2.285* [2.199,2.374]	1.679* [1.615,1.745]	1.899* [1.828,1.971]	1.291* [1.246,1.338]
Failure in treatment	0.786* [0.755,0.818]	0.858* [0.827,0.889]	0.792* [0.765,0.821]	0.826* [0.798,0.854]	1.103* [1.074,1.132]
Female staff not available		0.333* [0.288,0.385]	0.682* [0.613,0.758]	0.322* [0.279,0.373]	2.086* [1.937,2.246]
Other staff uncooperative					0.397* [0.339,0.466]
Other complains	4.072* [3.767,4.401]	2.494* [2.301,2.702]	1.935* [1.781,2.103]	1.735* [1.597,1.886]	1.522* [1.402,1.652]
Observations (all households where a mother visited a healthcare facility)	2562	2580	2580	2580	2592

Note: *p < 0.05.

Table 4 presents the national-level analysis of MOV, including all socioeconomic and demographic factors among the children whose mothers visited a healthcare facility. Urban residence, smaller households, and those with unemployed mothers were associated with higher odds of missing all doses of vaccines compared to rural areas. Male children had marginally lower odds of missed opportunities for early vaccinations, but higher odds for Measles-2 (OR: 1.149, CI: 1.133,1.165) than female children.

The model also accounted for complaints about healthcare facilities, with uncooperative staff, long waiting times, non-availability of medicine, and lack of cleanliness notably associated with higher odds of missed opportunities for all vaccines. The wealth index did not exhibit a linear trend indicative of lower odds of missing vaccines among wealthier households compared to the poorest quintile.

## Discussion

Missed vaccination remains a significant public health concern in Pakistan. Our findings highlighted that one in six children missed the third dose of the pentavalent vaccine and one in four missed the second dose of the measles vaccine. Missed doses were more common in districts with sparse populations, perhaps reflecting geographical remoteness. Our central hypothesis was that maternal visits to healthcare providers offer an underutilized opportunity to catch up on children’s vaccinations. However, these opportunities are often missed. MOVs were more likely in households that were urban, male-led, poorer, had fewer children (younger mothers), and where mothers had lower education or were not employed. Male children were also more likely to be missed. These findings are consistent with global experience. ^24^ Similar issues have been observed for other neglected groups, such as urban slums, which may present a special issue in that many such communities are underreported and therefore underserved with government services such as immunization, ^25^^,^^26^ for transient migrant populations, or hard-to-reach rural communities. ^27^^–^^29^

These household-level determinants intersect with broader systemic patterns in healthcare utilization, particularly the division between public and private sectors. In Pakistan, nearly all vaccinations are administered in the public sector, yet only 19% of healthcare visits take place there. ^30– 34^ Private healthcare use is especially dominant in urban settings. while missed doses are more prevalent in remote and rural areas (due to limited supply and infrastructure), ^8^ MOV are more frequent in urban locales, not because of vaccine availability, but because care is typically sought in the private sector, which rarely provides immunization services. The PSLM data do not include information on whether private clinics offer vaccines, so while we observe an association, we cannot infer causality. This highlights the risk of ecological fallacy and the need for caution in interpreting urban-private MOV patterns.

In Pakistan, and many LMIC, the private sector consists largely of small single-provider clinics that operate at low margins. ^35^ These clinics often lack immunization supplies, contributing to MOVs. However, this also presents an opportunity: if these maternal visits can be used to refer unvaccinated children to nearby public facilities, vaccination coverage could significantly improve. These clinics often lack immunization supplies, contributing to MOVs. However, this also presents an opportunity.

In addition to systemic interventions, household-level dynamics also shape vaccination outcomes. Household structure also plays a crucial role in vaccination coverage. Children in larger families (with multiple caregivers and stronger social networks) were less likely to experience missed doses. ^36, 37^ Mothers who are educated, employed, or lead their households appear to exercise greater agency in ensuring children receive vaccines. ^38– 40^ These findings reinforce the value of empowering mothers and promoting their role in healthcare decisions. ^41^

MOVs, as demonstrated in our study, are driven by both systemic factors (e.g., public-private fragmentation, supply gaps) and household-level characteristics (e.g., maternal education, poverty, family structure). Tackling these requires multi-level interventions. Structural reforms such as public-private integration and referral systems must be paired with community-level behavior change strategies to empower mothers and caregivers.

This pattern supports the interpretation that these children are not lost due to household disengagement, but because of persistent gaps in system follow-up. This is further supported by our findings that the dropout rate across first, second, or third doses remain constant, suggesting that these households are missed by the health system rather than due to any household factors. This is consistent with the global experience that links low coverage with healthcare system inefficiencies, including financial constraints, vaccine stockouts, staff absenteeism, and negative attitudes such as misconceptions about vaccinating sick children, that significantly contribute to high MOVs. ^23^^,^^42^^–^^44^ This suggests that increasing the capacity or agency of mothers with prompts and nudges to demand vaccination for their children when they visit healthcare providers may improve vaccination rates considerably, irrespective of other factors. ^45^^–^^48^ Finally, robust accountability frameworks will further align efforts with Pakistan’s Expanded Program on Immunization (EPI) goals. ^49^^–^^51^

### Limitation and Future Policy Implications

This study relied on existing national survey data, over which the investigators had no control regarding question wording or recall periods. In particular, maternal healthcare visits were captured only for the two weeks preceding the survey, which may have led to an underestimation of MOV. In addition, the analysis could not account for certain clinical and service-level factors, such as illness severity during maternal visits, provider referral practices, or recall bias in vaccination history. These unmeasured factors may have influenced the observed associations and warrant cautious interpretation of the findings.

The scope of the analysis was further shaped by data limitations related to vaccine delivery mechanisms. Although Pakistan continues to face challenges in polio eradication, polio vaccination was not included because the PSLM dataset does not capture information on supplementary immunization activities, door-to-door campaigns, or repeated oral polio vaccine doses. As polio delivery differs substantially from routine EPI vaccines, particularly pentavalent and measles, missed opportunities for polio vaccination cannot be reliably inferred from maternal healthcare visits. Consequently, the findings of this study should not be directly extrapolated to polio vaccination without dedicated data capturing campaign-based delivery.

Within these constraints, the policy recommendations emphasize interventions that are both evidence-informed and operationally feasible. Integrating vaccine screening prompts into digital health platforms used by private providers represents a high-feasibility, short-term intervention that leverages existing urban primary care infrastructure. Such systems can prompt providers to routinely assess a child’s immunization status during visits and refer eligible children to nearby government vaccinators, potentially yielding rapid gains in referral and reporting. ^52^ Community engagement initiatives to increase vaccine demand require sustained outreach and coordination and are therefore more appropriately positioned as medium-term strategies. In contrast, formal integration of private providers into national immunization planning constitutes a longer-term structural reform requiring regulatory alignment and institutional capacity building.

## Conclusion

To address the critical issue of missed vaccinations, there are considerable opportunities to catch up when a mother visits a healthcare provider for a medical visit. Availing these can help increase immunization rates by several percentage points and perhaps even help achieve the over 90% target of immunization in Pakistan. Therefore, we suggest a way to overcome the issue of vaccinations for households that avail healthcare from the private sector and include mothers in the process of managing their children’s vaccinations. Finally, using geospatial maps to identify high missed vaccination localities, especially concentrating on them, will also help improve vaccination rates. ^10^

## Data Availability

The PSLM 2019–20 dataset used in this study is publicly available through the Pakistan Bureau of Statistics website: https://www.pbs.gov.pk/content/pslm-district-level-survey-2019-20-microdata. No restrictions apply.
